# The cost of safe sex: estimating the price premium for unprotected sex during the Avahan HIV prevention programme in India

**DOI:** 10.1093/heapol/czz100

**Published:** 2019-10-11

**Authors:** Matthew Quaife, Aurélia Lépine, Kathleen Deering, Fern Terris-Prestholt, Tara Beattie, Shajy Isac, R S Paranjape, Peter Vickerman

**Affiliations:** 1 Department of Global Health and Development, London School of Hygiene and Tropical Medicine, London, UK; 2 Department of Medicine, Faculty of Medicine, University of British Columbia, Vancouver, Canada; 3 Gender and Sexual Health Initiative, BC Centre for Excellence in HIV/AIDS, St. Paul’s Hospital, Vancouver, Canada; 4 Monitoring and Evaluation, Research and Special Studies, Karnataka Health Promotion Trust, Rajajinagar, Bangalore, India; 5 Department of Community Health Sciences, Center for Global Public Health, University of Manitoba, Canada; 6 National AIDS Research Institute, Pune, India; 7 School of Social and Community Medicine, University of Bristol, Bristol, UK

**Keywords:** HIV prevention, condom use, instrumental variables, compensating differential, sex work, India

## Abstract

There is some evidence that female sex workers (FSWs) receive greater earnings for providing unprotected sex. In 2003, the landscape of the fight against HIV/AIDS dramatically changed in India with the introduction of Avahan, the largest HIV prevention programme implemented globally. Using a unique, cross-sectional bio-behavioural dataset from 3591 FSWs located in the four Indian states where Avahan was implemented, we estimate the economic loss faced by FSWs who always use condoms. We estimate the causal effect of condom use on the price charged during the last paid sexual intercourse using the random targeting of Avahan as an instrumental variable. Results indicate that FSWs who always use condoms face an income loss of 65% (INR125, US$2.60) per sex act compared to peers providing unprotected sex, consistent with our expectations. The main finding confirms that clients have a preference for unprotected sex and that policies aiming at changing clients’ preferences and at improving the bargaining power of FSWs are required to limit the spread of HIV.

**Key Messages**

- Using data from 3591 female sex workers (FSWs) located in four Indian states where the *Avahan* programme was implemented, we estimate the loss faced by FSWs who always use condoms.
- We use an instrumental variable to correct for endogeneity in the impact of condom use on act price.
- We find that FSWs who always use condoms face income losses of 78%, INR150 ($2.35) per act, compared to their peers who offer unprotected sex.

## Introduction

HIV prevalence in India is <1% in the general population, but female sex workers (FSWs) are up to 25 times more likely to be HIV positive than other women (Ramesh *et al.*, 2008). Many FSWs in India report violence and intimidation by clients and the police, with experience of violence strongly associated with inconsistent condom use and lower levels of participation in HIV and sexually transmitted infection (STI) prevention activities (Beattie *et al.*, 2010). In the context of heightened economic and structural inequities, FSWs are highly financially dependent on sex work to support themselves and their families (Bharat *et al.*, 2013; Dasgupta, 2013; Sahni and Shankar, 2013). As a result, FSWs often need to make difficult decisions regarding their own health, alongside ensuring economic and social stability for themselves and their dependents.

There is evidence in the economic literature that FSWs face a positive price premium for unprotected sex, defined as the *condom differential* (Rao *et al.*, 2003; Gertler *et al.*, 2005; de la Torre *et al.*, 2010; Robinson and Yeh, 2011; Arunachalam and Shah, 2013; Muravyev and Talavera, 2013; Cunningham and Kendall, 2014; Egger and Lindenblatt, 2015). In those studies, while the condom differential is always positive, it varies from a reduction in the price for protected sex of 81% (Bangladesh) to 7% (Belgium and Netherlands), though varying statistical methods make direct comparisons of this premium challenging.

In the face of a growing and predominantly heterosexually driven HIV epidemic, India experienced a major change in the fight of HIV/AIDS with the introduction of the largest HIV prevention initiative implemented globally: *Avahan*—the Hindi word for ‘welcome’. Avahan was introduced in 2003 in four Southern Indian states in order to address proximal and distal determinants of HIV risk. It delivered a comprehensive package of HIV prevention services including peer-education, STI treatment, condom promotion and distribution, and community mobilization to reduce social stigma (Ramakrishnan *et al.*, 2010). Non-governmental organizations (NGOs) working with key populations (FSWs, their clients and men who have sex with men) were provided grants by the Bill and Melinda Gates Foundation to provide those HIV prevention services. Many employed peer educators, current or former FSWs received training on HIV prevention and Avahan service delivery, before providing those services to an average of 25–50 persons at high risk of HIV. Peer educators shared prevention information, distributed condoms and lubricants, and provided referral for the management of STIs. Avahan achieved an exceptional scale-up of HIV prevention services, reaching 725 040 high-risk persons between 2004 and 2007, distributing 177 million condoms and conducting 529 381 STI tests (Ramakrishnan *et al.*, 2010; Verma *et al.*, 2010).

In this study, we estimate the economic loss faced by FSWs who always use condoms. We estimate the causal effect of condom use on the price charged during the last paid sexual intercourse using the random targeting of Avahan as an instrumental variable, analysing a cross-sectional survey dataset collected 28–37 months after the introduction of Avahan activities. This dataset is much larger than others in the economics literature, containing price data from over 3500 FSWs. In line with earlier studies, we use intervention exposure as an instrumental variable to correct for endogeneity bias, as a number of factors could affect both condom and price negotiation.

Table 1 shows that in the literature, the price premium for unprotected sex is highly context specific ranging from <10% in the USA (Cunningham and Kendall, 2016), Belgium and Netherlands (Adriaenssens and Hendrickx, 2012) and Kenya (Robinson and Yeh, 2011) to around 80% in India (Rao *et al.*, 2003) and Bangladesh (Islam and Smyth, 2012). While there have been several studies that aimed to measure the condom differential price, there is no evidence of the size of the condom premium in the context of intensive HIV prevention.

**Table 1.** Summary of selected economic studies estimating the condom differential

Authors	Date of publication	Setting	Number of Acts	Number of FSWs	Strategy to overcome endogeneity
Adriaenssens and Hendrickx	2012	Belgium and The Netherlands	25 000+	6400+	Fixed effects
Arunachalam and Shah	2013	Ecuador	8500	2800	Fixed effects
Cunningham and Kendall	2014	USA	2047	685	Fixed effects
de la Torre *et al.*	2010	Mexico	429	429	Paired price model
Egger and Lindblatt	2015	Germany	16 583	2517	Instrumental variables (prior average risk taking of other sex workers, and the average height of other sex workers)
Gertler, Shah and Bertozzi	2005	Mexico	∼4000	1029	Fixed effects
Islam and Smyth	2012	Bangladesh	283	283	Instrumental variable (participation in a safe sex training programme)
Manda	2013	Kenya	19 041	192	Fixed effects
Muravyev and Talavera	2015	UK	13 876	3877	Fixed effects
Rao *et al.*	2003	India	608	608	Instrumental variable (participation in safe sex training programme)
Robinson and Yeh	2011	Kenya	19 041	192	Fixed effects

Measuring the causal effect of condom use on prices charged for sex is challenging because many factors that may have a critical impact on both price are condom use are often not measured in surveys and will confound the effect. For example, those factors may include unobserved characteristics of a FSW and her client that might influence both the price bargaining and condom bargaining, such as negotiation skills, past experiences during negotiation (e.g. violence), drug use and the immediate need for cash. Even when observed, the reliability and validity of some factors can often be questioned. Therefore, simply regressing the price charged for sex on condom use will fall short in estimating a causal effect of condom use on price, and result in a biased estimate; this is the endogeneity problem.

There have been many attempts in the literature to overcome the endogeneity problem in estimating the impact of condom use on price charged for sex. Table 1 shows that published studies either used a fixed effect estimator that accounts for time-invariant unobserved characteristics of FSWs or an instrumental variable that induces an exogenous variation in condom use in order to overcome endogeneity. Evidence from studies that used an instrumental variable approach highlighted that the endogeneity bias resulted in an underestimation of the effect of condom use on price, suggesting that omitted variables may be positively correlated with condom use and the price charged (e.g. bargaining power).

## Methods

### Data and study setting

In 2003, Avahan was implemented among FSWs and other key populations Activities included condom promotion, STI management and creating an environment where safe sex was socially accepted (Chandrasekaran *et al.*, 2008). The intervention aimed to reach 80% of all high-risk groups, including FSWs and their clients. To assist in the evaluation of the programme, several cross-sectional bio-behavioural surveys [Integrated Behavioural and Biological Assessments (IBBAs)] were carried out, and information on the construction and implementation of the programme and IBBA surveys has been widely published elsewhere (Ramesh *et al.*, 2008; Saidel *et al.*, 2008; Deering *et al.*, 2011; Pickles *et al.*, 2013). The IBBAs aimed to inform a population-level analysis of Avahan in a causal-pathway-based modelling analysis (Pickles *et al.*, 2013), were carried out as face-to-face interviews using a culturally sensitive and context-specific questionnaire translated into the local languages, alongside the collection of blood samples.

We use cross-sectional data gathered between 2006 and 2009, between 28 and 37 months after the start of Avahan. As described in detail elsewhere (Saidel *et al.*, 2008), data were gathered via culturally sensitive and context-specific questionnaires from 3591 FSWs sampled through a two-stage cluster process in 118 districts in four states; Andhra Pradesh, Karnataka, Maharashtra and Tamil Nadu. Data were available for two other states (Manipur and Nagaland); however, due to a substantively different epidemic context, specifically the prevalence of injecting drug use, we elect not to include this state in this analysis.^1^ Sampling weights are used in this analysis for each district, proportional to the number of FSWs estimated to sell sex in the district and typology of FSW.

### Empirical model

We use a standard two-stage least squared (2SLS) approach to correct for endogeneity in estimating the effect of condom use on price. The first stage estimates:

(1) $${\hat{C}}_{i}=\;\delta_{0}+\delta_{1}X_{i}+\;\delta_{2}Z_{i}+r_{i},$$

where ${\hat{C}}_{i}$ is a binary outcome variable denoting consistent condom use and coded 1 if a FSW reported using condoms *all of the time* with occasional and regular clients, and 0 otherwise. Note that the IBBAs did not collect data on types of sex act (e.g. vaginal, oral or anal), so we are not able to explore this variation in condom use. $X_{i}$ a vector of FSW characteristics, $Z_{i}$ our instrument and $r_{i}$ the error term. We define our instrument as whether FSWs obtained their last condom from an *Avahan* peer educator/outreach worker. All respondents were asked where the last condom was obtained from. We code the subsequent variable 1 if the answer is a peer educator/outreach worker (75%) or NGO outreach van (6%), and 0 if any other answer. Those answers include shop (1%), pharmacy (3%), client (4%), hospital (1%), bar (4%), brothel madam (2%) and other (4%). Note that the high proportion who received a condom from Avahan is high (81%) because of the large size of this prevention project. In fact, all FSWs located in an Avahan state were at least reached once by Avahan. For brevity, we refer to this as the ‘last condom’ instrument. We argue that this instrument does not violate the exclusion criterion as condom distribution depended entirely on outreach worker workload and not on the characteristics of FSWs to whom condoms were distributed. We explore this intuition further in robustness checks in the Results section.

We substitute ${\hat{C}}_{i}$ into:

(2) $$P_{i}=\;\beta_{0}+\beta_{1}X_{i}+\;\beta_{2}{\hat{C}}_{i}+\varepsilon_{i,}$$

where *P_i_*denotes the price a FSW charged in the last commercial act. $\varepsilon_{i}$ is the error term.

### Variable specification

$X_{i}$ contains a number of relevant covariates. First, we consider the type of alternative employment available to FSWs as this may affect the quantity and type of sex she needs to sell to earn a living (Reed *et al.*, 2010; Mccarthy *et al.*, 2014). Second, the environment where FSWs entertain clients, e.g. a secure indoor setting vs a less-safe street setting, can affect price (Dandona *et al.*, 2005; Shannon *et al.*, 2009). Third, having young dependents (we define as children under 5 years of age) has been shown to be negatively associated with price (Evans and Lambert, 2008; Papworth *et al.*, 2015). Fourth, we consider FSW community cohesion proxied by a variable denoting whether or not a woman feels a strong sense of unity with other FSWs. Finally, we include recent experience of violence (in the past 6 months); this has been associated with a reduced likelihood of condom use in commercial acts, particularly where sex work is criminalized and FSWs subsequently operate in more insecure, isolated spaces (Deering *et al.*, 2013). Violence towards FSWs can be enabled by unsupportive institutions, often signals isolation or disempowerment of the FSW community, and substantively impacts negotiation between FSWs and clients (Siegfried *et al.*, 2003; Shannon and Csete, 2010; Shannon *et al.*, 2015).

Characteristics of women in sex work that objectively appeal to all clients, including various physical or personality features, are difficult to measure. The economic literature has typically simplified these features by attempting to capture the physical attractiveness of FSWs or clients (Arunachalam and Shah, 2012; Islam and Smyth, 2012). Outside of sex work, there is some evidence from labour economics that attractiveness is associated with better bargaining outcomes, more generous treatment and greater cooperation in negotiation (Mulford *et al.*, 1998; Solnick and Schweitzer, 1999; Rosenblat, 2008). Ultimately, these physical or personality traits influence the price charged by FSWs. The IBBA surveys do not contain direct measures of attractiveness, so we created a set of proxy variables that encompass multiple features of bargaining power and acknowledge economic work on the influence of physical features: FSW education (a binary variable if the FSW is literate); the number of children she has, the ratio of her age divided by her tenure in sex work (in years) and whether she is currently married. We control for a FSW’s use of non-condom forms of contraception including oral pills, IUDs, injections and sterilization.

We log-transform the dependent variable price, *P_i_*, due to it being right-skewed. Where interpreted, the effect of the binary condom use variable on price is calculated using Giles' method (1982):

(4) $$\%\Delta \left(\mathrm{Price}\right)=100\left(e^{\beta_{2}}-1\right),$$

where $\beta_{2}$ is the condom use coefficient.

## Results

### Descriptive statistics

Data were from 3591 FSWs from 118 districts in four states and Table 2 summarizes key descriptive statistics. The median price per act in the sample was INR200 (US$4.13); although 90% of FSWs charged less than INR500 ($10.33), the maximum recorded figure (INR4000 US$82.66) was 20 times the median. On average, FSWs worked 4.3 days per week and had 2.6 clients per day. Mean daily earnings from sex work were around INR700 (US$11), over 6 times greater than the average daily wage for a non-salaried Indian woman of around INR110 (US$2.27) (Ministry of Statistics and Programme Information, 2013).

**Table 2.** Descriptive statistics of study population (*n* = 3591)

Variable	Mean/proportion	SD
Price (2006 INR)	270 (median: 200)	235 (IQR: 100–350)
Ln(Price)	5.31	0.753
Consistent condom use with all clients	0.87 (3124)	0.343
Literate	0.39 (1400)	0.489
Age (years)/time as sex worker (years)	9.56 (34 330)	7.88
Children (number)	1.85 (6643)	1.16
Currently married	0.63 (2262)	0.484
Using non-condom contraception	0.61 (2191)	0.489
Feels strong sense of unity with other FSWs	0.86 (3088)	0.343
Has a child under 5 years old	0.27 (970)	0.445
Experienced violence in past 6 months	0.16 (575)	0.364
HIV positive	0.16 (575)	0.363
Sex work environment		
Home	0.27 (970)	0.444
* *Rented room	0.25 (898)	0.436
* *Lodge	0.21 (754)	0.406
* *Dabha (truck stop)	0.002 (7)	0.045
* *Brothel	0.16 (575)	0.370
* *Bar/nightclub	0.001 (4)	0.036
* *Public place	0.01 (36)	0.299
* *Other	0.1 (359)	0.016
Other employment		
* *None	0.49 (1760)	0.5
* *Non-agricultural labour	0.19 (682)	0.395
* *Petty business	0.07 (251)	0.25
* *Maid/servant	0.11 (395)	0.318
* *Agricultural labour	0.1 (359)	0.295
* *Artisan/handicrafts	0.03 (108)	0.183
State		
* *Andhra Pradesh	0.2 (718)	0.393
* *Karnataka	0.28 (1005)	0.447
* *Maharashtra	0.29 (1041)	0.453
* *Tamil Nadu	0.22 (790)	0.415
Last condom obtained from Avahan	0.82 (2945)	0.385

The median age of the sample was 31 years (interquartile range (IQR): 26–38) and average duration in sex work was 4 years (IQR: 2–8). The median number of regular and occasional clients in the last 10 days were 7 (IQR: 5–8) and 3 (IQR: 2–5), respectively. Although the median age of entry into sex work was 25 years (IQR: 21–31), 287 (8%) report beginning sex work under the age of 18. A large proportion (3303, 92%) of FSWs reported having children, and the median number of children of women in the sample was 2 (IQR: 1–3). Overall, 1400 (39%) of participants reported being able to read and write, 2262 (63%) were married and 2190 (61%) reported using non-condom contraception. Finally, 3124 (87%) of women in the study reported consistent condom use with all clients. We restrict our sample to those who have been reached by Avahan, with the mean length of time since first contact of 140 weeks. Table 2 shows the variable used to construct our instrument, where a FSW obtained her last condom, with 2944 (82%) of women obtaining their last condom from a peer/support worker or NGO van.

### Econometric results

Table 3a presents ordinary least squares (OLS) model estimates. This specification suggests condom use has a small and non-significant negative effect on price (*P*-value = 0.72). Table 3b(i) displays our main model, an instrumental variable (IV) estimation of equation (2) using the last condom instrument in the 2SLS regression. Table 3b(ii) gives the first-stage output, equation (1). There is strong evidence (*P *< 0.05) that condom use is negatively associated with price per act, and on average a FSW who always uses condoms will face income losses of 65%, or INR125 ($2.60) per act compared to her peers who offer unprotected sex, which is close to the 79% obtained in Rao *et al.* (2003). The difference between OLS and IV estimates suggests that, as anticipated, not controlling for omitted variable bias in the OLS specification leads to the underestimation of the effect of condom use on price. Furthermore, we note that the magnitude and direction of other variables do not change substantially between the two specifications.

**Table 3.** OLS and IV results estimating the condom differential

	(a) OLS		(b) 2SLS		(c) First stage of (b)		(d) 2SLS		(e) 2SLS	
	ln(price)	SE	ln(price)	SE	Consistent condom use		ln(price)	SE	ln(price)	SE
Instrument: Last condom	No		Yes				No		Yes	
Instrument: Number of NGO contacts	No		No				Yes		Yes	
Consistent condom use with all clients	−0.017	0.065	−1.04**	0.43			−1.78**	0.89	−1.19***	0.40
Literate	0.32***	0.044	0.36***	0.039	0.033**	0.013	0.38***	0.054	0.36***	0.041
Age (years)/time as sex worker (years)	0.0029	0.0019	0.0044*	0.0022	0.0015	0.0013	0.0072**	0.0035	0.0058**	0.0026
Children (number)	−0.0090	0.019	−0.034	0.022	−0.023***	0.0070	−0.048*	0.028	−0.034	0.022
Currently married	0.16***	0.043	0.16***	0.036	0.0096	0.013	0.17***	0.044	0.16***	0.039
Using non-condom contraception	−0.010	0.054	0.0057	0.041	0.0085	0.029	0.012	0.052	0.0057	0.044
Feels strong sense of unity with other FSWs	0.040	0.074	0.11*	0.059	0.050	0.047	0.14	0.089	0.11*	0.064
Has a child under 5 years old	0.071	0.041	0.065	0.041	−0.0039	0.024	0.087	0.054	0.088**	0.044
Experienced violence in past 6 months	0.032	0.053	−0.074	0.069	−0.098***	0.030	−0.17	0.12	−0.10	0.074
HIV positive	−0.15**	0.052	−0.14***	0.047	0.0097	0.018	−0.15**	0.060	−0.15***	0.051
Other employment:										
* *None										
* *Non-agricultural labour	−0.073	0.080	−0.15**	0.064	−0.079	0.049	−0.20**	0.096	−0.16**	0.067
* *Petty business	−0.020	0.092	−0.068	0.089	−0.058	0.038	−0.12	0.10	−0.093	0.091
* *Maid/servant	0.054	0.078	−0.0004	0.063	−0.053	0.044	−0.052	0.087	−0.020	0.065
* *Agricultural labour	−0.32**	0.12	−0.29***	0.083	0.039	0.033	−0.28***	0.11	−0.30***	0.088
* *Artisan/handicrafts	0.15	0.11	0.11	0.094	−0.032	0.031	0.044	0.12	0.072	0.10
States										
* *Andhra Pradesh										
* *Karnataka	0.0023	0.22	−0.079	0.066	−0.072	0.047	−0.15	0.11	−0.099	0.072
* *Maharashtra	−0.43***	0.11	−0.36***	0.081	0.092**	0.040	−0.34***	0.11	−0.37***	0.083
* *Tamil Nadu	0.35***	0.087	0.47***	0.093	0.12***	0.034	0.57***	0.13	0.49***	0.093
Last condom Avahan					0.12***	0.027				
Constant	5.15***	0.13			0.78***	0.055				
Observations	3581		3581		3581		3581		3581	
*R* ^2^	0.3		0.03		0.119		0.336		0.017	
Kleibergen-Paap rk Wald *F* statistic			18.537				5.879		10.08	

Robust standard errors. For presentation due to partialling out, coefficients for place of entertainment in (a) are not shown but were included in the model. The Stock-Yogo weak ID Test 10% and 15% critical values are 16.38 and 8.96, respectively. First-stage results of model (d) shown in Supplementary Table S1.

*** *P* < 0.01.

** *P* < 0.05.

* *P* < 0.1.

While not the main focus of the article, the results highlight that other factors affect prices charged for sex work. Prices are higher for FSWs who are literate (*n* = 1485, 39%), or currently married (2343, 62%), but lower for those who are HIV positive (588, 16%); findings consistent with the economic literature on attractiveness. There is some evidence (*P* = 0.07) that feeling a strong sense of unity with other FSWs is associated with a higher price charged.

As a first robustness check, we use an alternative indicator of Avahan exposure as instrument: *number of contacts from an Avahan-supported NGO in the last 6 months*. Table 3(d) displays the results of using just this instrument, and Table 3(e) the over-identified specification with both instruments; both show consistent results for the coefficient of interest, and the effect of covariates on price. We apply the Sargan test (Sargan, 1958) to the over-identified model and are unable to reject the null hypothesis that both instruments are valid.

### Instrument validity

It is simple to test the explanatory power of our instrument on condom use, and we find it exceeds the conventional benchmarks of *F* > 10 in the first stage (*F* = 10.26), whilst the Kleibergen-Paap rk Wald *F* statistic (18.54) performs well against Stock-Yogo critical values exceeding the 10% maximal IV size (Stock and Yogo, 2005).

While it is not possible to test if the instrument does not violate the exclusion restriction, we question whether FSWs who received their last condom from Avahan were not systematically different than those who did not receive their last condom from Avahan. To assess the likelihood of a particular peer outreach worker contacting a FSW, we extensively examined the strategy outreach staff used to reach women. Peer outreach workers had a responsibility to reach and engage with every FSW operating in an allocated geographic area, whilst we note that this was not dependent on the place of work since Avahan targeted all types of FSWs (including street-based, bar-based or brothel-based FSWs). Areas were assigned by NGOs annually and each peer outreach worker had around 50 FSWs in her network. Using key-informants and their own network, peer outreach workers aimed to regularly identify new FSWs as well as contact each FSW in her area every 15 days; it was during these contacts that condoms were distributed (Chandrashekar, 2014, personal communication). We argue that, because peer support workers were required to contact every FSW regularly, the probability a FSW was contacted (and condoms distributed) recently before they were surveyed is independent of any of her characteristics, except perhaps the peer outreach worker’s workload.

Alongside condom distribution, Avahan also provided information on HIV, STI and pregnancy prevention options. It is plausible that increasing FSWs’ knowledge of the potential consequences of unprotected sex will increase the premium required to adequately compensate for the risk of unprotected intercourse. If a proportion of the population was non-randomly chosen to receive information (or supplies of free condoms), the exclusion restriction would not be satisfied and our results biased. Because all of the sample was reached at some point by Avahan, we assume that knowledge of the risks of unprotected sex is uniform across our sample and will not directly influence price according to FSW characteristics.

Table 4 explores if there are systematic differences between FSWs who did and did not receive their last condom from Avahan, i.e. when $Z_{i}$ equals 1 and 0, respectively. We test for differences between factors included in the models above, except those which Avahan explicitly --aimed to change since data come from after programme implementation (using non-condom contraception and feeling unity with other FSWs). In addition, we test for differences in five other indicators which may signal greater or lesser FSW risk-taking behaviours. These results generally support the argument that condoms were distributed with little regard for FSW characteristics, though we find that FSWs receiving their last condom from a peer educator were more likely to be literate and less likely to be married.

**Table 4.** Differences between FSWs receiving and not receiving last condom from Avahan

Variables	Did not receive last condom from Avahan	Received last condom from Avahan	*t*-test *P*-value
Price of last act	273.35	266.23	0.50
Literate	0.35	0.41	0.01
Age (years)/time as sex worker (years)	9.42	9.58	0.60
Children (number)	1.81	1.86	0.40
Currently married	0.66	0.61	0.02
Has a child under 5 years old	0.28	0.26	0.40
Age of sexual debut	15.96	15.92	0.70
Age of sex work debut	25.89	26.15	0.30
Ever provided anal sex to a client	0.13	0.13	0.96
Average number of clients per week	11.86	12.21	0.50
Regularly consumes alcohol	0.11	0.10	0.60

Nb, numbers are adjusted by sample weights.

## Discussion

We used Avahan outreach as an instrumental variable to investigate the effect of using condom on the price charged in India. We estimated that FSWs who provide protected intercourse face income losses 65% or INR125 (US$2.60) per act provided compared to their counterparts offering unprotected sex act.

Evidence suggests that the Avahan programme has been successful in reducing the HIV epidemic and is estimated to have prevented up to 600 000 infections over the decade since its introduction in 2003 (Chandrashkar *et al.*, 2011; Pickles *et al.*, 2013). Despite this, our study suggests that more than 4 years after the initiation of Avahan, FSWs continued to face heightened vulnerability to HIV and STIs through economic pressures. Explanations for the persistence of the condom differential in India can be found through exploring both the supply and demand sides to understand the intersecting vulnerabilities faced by FSWs.

Firstly, the price premium for unprotected sex may have been high 4 years into the Avahan intervention if the programme constrained the supply of unprotected sex by reducing the number of FSWs who agree to client demands for unprotected sex. If client demand remained constant, or fell at a lower rate than the supply of unprotected sex, remaining FSWs who agreed to sex without a condom would be able to command higher prices.

Secondly, FSWs may agree to better-compensated condomless sex because of unpredictable income fluctuations. Programmes which aim to reduce the economic vulnerability of women, such as encouraging lending through formal microfinance or banking systems (which are often denied to FSWs due to stigmatizing structural institutional practices) may limit FSW need to agree to sex without a condom (Evans and Lambert, 2008), or reduce dependence on informal lending (Sherman *et al.*, 2010).

Thirdly, our results suggest that FSWs who feel unity with colleagues in sex work may be able to negotiate higher prices, supporting the focus of many FSW programmes on supporting community empowerment, e.g., enabling women to work collaboratively by facilitating the development of policies surrounding condom use that are collectively supported (Halli *et al.*, 2006; Guha *et al.*, 2012; Kerrigan *et al.*, 2015). A major barrier to the development of safer sex work spaces is the current legal framework surrounding sex work, where working in indoor, organized settings is criminalized, including in southern India. Decriminalization of sex work is therefore crucial to address in order to support FSWs’ agency in insisting on condom use by clients (Quast and Gonzalez, 2016; Cunningham and Shah, 2017).

Finally, recent advances in the efficacy of bio-medical interventions, which reduce the risk of contracting HIV in the absence of condoms, are starting to change the landscape of HIV prevention (AVAC, 2015). In addition, HIV treatment continues to be scaled-up, decreasing the number of people living with HIV who are likely to be infectious (National AIDS Control Organisation, 2011) although implementing this in the sex work context is likely to be challenging. Introducing treatment or prevention methods may be seen as exogenous shocks to the market for commercial sex, yet many trials and implementation programmes do not collect data on market or pricing dynamics. Our results indicate that HIV prevention programmes do not occur in a vacuum and may have indirect but important influences on the preferences and incentives of FSWs and clients.

We acknowledge that our study has some limitations. As in any application of instrumental variable methods, we are unable to test whether our instrument operates in violation of the exclusion restriction. However, through exploring intuition and testing where possible, we showed that our last condom instrument convincingly satisfies the relevance and exogeneity conditions. One difficulty in interpreting the estimates of IV methods is that not everybody responds in the same way to exposure to the instrument (Angrist and Krueger, 2001). For example, there may be a subset of FSWs who would always (or never) provide protected intercourse, whether or not they are contacted by Avahan or receive free condoms.

We acknowledge that the IV method may not eliminate all sources of bias. In this study, we are only able to estimate the premium for unprotected intercourse for FSWs whose choice of using condom has been influenced by receiving their last condom from Avahan. Although data were captured on condom use for regular and occasional clients, pricing data were not available for each group and we were therefore unable to decompose our premium estimate by client type. Because protection and pricing dynamics may be substantially different with different clients, it would be useful for further work to explore this. Finally, because information on condom used is sensitive and self-reported by FSWs, our results are susceptible to acceptability biases.

Finally, because of a range of factors, including gender-based social inequities and social stigmatization of sex work, few studies of clients have been conducted. Gaining a better understanding of why clients demand unprotected sex, and how this demand can be mitigated through HIV prevention programming, is critically important to reducing HIV risk to FSWs and the overall HIV epidemic in southern India.

## Conclusion

We applied instrumental variable method to a large dataset of FSWs in India to estimate the size of the price premium for unprotected sex, 4 years following the initiation of the Avahan HIV prevention intervention. This study finds that a premium for unprotected sex is almost identical to that found in previous work in India before Avahan. After correcting for endogeneity bias, we conclude that FSWs in southern India who report consistent condom use by clients experienced a loss of income of 65% per sex act. Given the uncertainty regarding how HIV treatment and prevention programs may affect the commercial sex market, we recommend that such programs explicitly consider the critical role that social, structural and economic vulnerability can play in FSW agency, and specifically decision making for protected intercourse.

## Supplementary Material

czz100_Supplementary_Table_1

## Footnotes

1 We note, however, that analysis of these states indicated similar results to those included in this study, results available upon request.
