# Supplementary material for: The cost of safe sex: estimating the price premium for unprotected sex during the Avahan HIV prevention programme in India
Source: Health Policy Plan. 2019 Oct 11;34(10):784–91. doi: 10.1093/heapol/czz100 (PMC6913694; doi:10.1093/heapol/czz100)
Supplement: czz100_Supplementary_Table_1 [file czz100_supplementary_table_1.docx]

**Supplementary table 1: First stage regressions for model 3(d)**

|  | (a) First stage of model 3(d) |  |
| --- | --- | --- |
| *Dependent variable* | *Condom use with last client* | |
| Literate | 0.031 | (0.19) |
| Age (Years) / Time as sex worker (Years) | -0.048*** | (0.016) |
| Children (Number) | -0.097 | (0.068) |
| Currently married | 0.12 | (0.16) |
| Using non-condom contraception | -0.51* | (0.25) |
| Feels strong sense of unity with other FSWs | -0.0093 | (0.43) |
| Has a child under 5 years old | -0.35** | (0.13) |
| Experienced violence in past 6 months | 0.60* | (0.29) |
| HIV positive | -0.10 | (0.23) |
| Other employment: |  |  |
| *None* |  |  |
| *Non-agricultural labour* | 0.013 | (0.28) |
| *Petty business* | 0.53 | (0.53) |
| *Maid/servant* | 0.060 | (0.27) |
| *Agricultural labour* | -0.073 | (0.60) |
| *Artisan/handicrafts* | 1.63 | (1.30) |
| State: |  |  |
| *Andhra Pradesh* |  |  |
| *Karnataka* | -1.87* | (1.02) |
| *Maharashtra* | -3.17*** | (0.88) |
| *Tamil Nadu* | -3.02*** | (1.00) |
| Number of NGO contacts | 0.11 | (0.33) |
| Constant | 6.43*** | (1.19) |
| Observations | 3,581 |  |
| R-squared | 0.152 |  |

*** p<0.01, ** p<0.05, * p<0.1.
